# Psychometric validation of the Cystic Fibrosis Impact Questionnaire (CF-IQ): A patient-reported outcome assessing impacts of cystic fibrosis

**DOI:** 10.1371/journal.pone.0317775

**Published:** 2025-01-24

**Authors:** Daniel Serrano, Alyssa Uzumcu, Maya Gerstein, Nicolai D. Ayasse, Ella Engstrom, Frederick B. Barnes, Charles Iaconangelo, Teja Thorat, Lisa J. McGarry, Isabelle Sermet-Gaudelus

**Affiliations:** 1 Pharmerit International–An OPEN Health Company, Bethesda, MD, United States of America; 2 The Psychometrics Team, Sheridan, WY, United States of America; 3 Vertex Pharmaceuticals Incorporated, Boston, MA, United States of America; 4 INSERM U1151, Université Paris Cité, Centre de Références Maladies Rares Mucoviscidose et Maladies Apparentées, Hôpital Necker Enfants Malades, Paris, France; Cincinnati Children’s Hospital Medical Center, UNITED STATES OF AMERICA

## Abstract

The Cystic Fibrosis (CF) Impact Questionnaire (CF-IQ) was qualitatively developed to assess the impact of CF in the context of treatment advancements and increased longevity. This study reports the CF-IQ validation. In this noninterventional validation study, people with CF completed the 40-item CF-IQ and validating patient-reported outcome measures (PROMs) via electronic diaries at enrollment (baseline) and at the 4-week follow-up. Validation consisted of modern methods and focus groups to finalize structural validity, and classical methods to assess internal consistency [1–3], test-retest reliability [4,5], concurrent validity [5], and known-groups validity [5] of the CF-IQ. At baseline, 214 adults completed the survey; 193 completed the follow-up survey. Unidimensional item response theory (IRT) models were separately fit to 5 prespecified domains (Control and Burden of CF Treatment Impacts, Physical Activity Impacts, Social Activity Impacts, Emotional Impacts, and Work/School Limitation Impacts). IRT local dependence (LD) statistics identified 17 redundant items. Two independent CF-patient focus groups (14 total patients) confirmed these findings, and the 17 items were dropped. Each domain defined on the final 23 items achieved the criterion of exact model fit as measured by the root mean squared error of approximation (RMSEA, values = 0), Internal consistency (Cronbach’s α) values ranged from 0.81 to 0.89, 4 of 5 domains achieved acceptable test-retest reliability, with intraclass correlation coefficient (ICC) values ≥ 0.7, acceptable concurrent validity was achieved for all domains, and known-groups validity was established. The novel CF-IQ is a psychometrically robust PROM capturing patient-centric impacts of CF in the context of the current standard of care.

## Introduction

Cystic fibrosis (CF) is a life-shortening autosomal recessive disorder that impacts >80,000 people worldwide [6–8]. CF affects multiple organ systems, including the respiratory, gastrointestinal, and reproductive systems, due to mutations in the gene encoding the CF transmembrane conductance regulator (CFTR) ion channel [7,9] Consequent to this genetic mutation, CFTR dysfunction results in abnormal transport of salt and water across epithelial cell membranes throughout the body, causing thickening of mucus secretions [7,9]. This results in mucus retention, bronchial inflammation and infection, and finally, progressive lung damage and premature death [7,9].

Several patient-reported outcome measures (PROMs) have been developed to measure symptoms, impacts, and quality of life (QoL) in CF [10–12]. However, the majority of these PROMs were developed nearly two decades ago [13,14] when people with CF rarely survived to middle age due to more severe disease complications. Recent improvements in standards of care and widespread use of CFTR modulators (CFTRm) have increased life expectancy and QoL in CF [7,9].

This increase in longevity and corresponding expansion of life experiences motivates the development of new, reliable, and sensitive CF-specific instruments. Indeed, patients clearly express that new items are necessary to capture their experience [15]. The Cystic Fibrosis Impact Questionnaire (CF-IQ) was developed following current best practices and PROM development guidelines for use in an increasingly healthy CF population [16–18]. Impacts assessed are those that can only occur in individuals surviving into adulthood; for example, impacts related to advanced education, work and careers, and family. The CF-IQ complements existing CF-specific PROMs by capturing the broader impact of CF on peoples’ lives and characterizing their experiences as well as changes in their QOL in response to therapy [16,18,19]. In the content development of the CF-IQ, concept elicitation and cognitive interview debriefing with CF patients were employed, resulting in a draft 40-item questionnaire with 5- to 7-point verbal rating scales and a specified 7-day retrospective recall period [16]. Readers interested in the qualitative development can refer to McCarrier et al., 2020 for full details [10]. The objective of the present study was to complete the psychometric validation of the CF-IQ.

## Methods

### Overall study design, participants, and analysis

This noninterventional validation study (NIVS) consisted of 2 parts: part 1) a two-stage, mixed-methods analysis to examine and resolve redundant items in the qualitative development of the CF-IQ; part 2) a classical reliability and validity assessment of the final questionnaire items. The steps taken to validate the CF-IQ are shown in Table 1.

**Table 1:** Steps taken for CF-IQ validation.

1. Participant recruitment
2. Data collection via QuestionPro web-based survey
3. Data analysis
4. Part 1: CF-IQ item reduction (MPMs and focus groups)
5. Part 2: Scoring assessment and classical methods
6. Validated CF-IQ with reduced number of items

CF-IQ, Cystic Fibrosis Impact Questionnaire; MPMs, modern psychometric methods.

Individuals from the Rare Patient Voice (RPV) CF patient panel [20] were enrolled into this NIVS from 5 February 2020 through 13 March 2020, with the same participants involved in both part 1 and part 2 of the study. Eligible individuals were ≥18 years of age, living in the United States, had a CF diagnosis, were able to read, write, and speak English, were able to provide an estimate of their most recent percent predicted forced expiratory volume in 1 second (ppFEV_1_), were willing to complete the retest assessment, and were not participating in any interventional studies of CFTRm. The admissible ppFEV_1_ value range was between 15 to 130. Informed consent was obtained in writing from all individual participants included in the study via an electronic informed consent form prior to study enrollment and any data collection. This study, including all NIVS and focus group data-collection activities, were reviewed and granted an exemption for IRB oversight by the Advarra centralized institutional review board on November 13, 2019.

Between January and April 2020, enrolled participants completed the 40-item CF-IQ and validating PROMs at baseline and at follow-up (4 weeks later) (Table 2). Baseline for this NIVS, as with others, was simply the first data collection. Follow-up was conducted 4-weeks later to comply with the US food and drug administration’s (FDAs) division of clinical outcome assessment (DCOA) recommended retest interval for test-retest reliability. Demographic and clinical characteristics were gathered at enrollment. All data were remotely collected using the QuestionPro Survey environment, which minimized the effects of the SARS-CoV-2 pandemic on the conduct of this study. In addition to the validation analyses (MPMs, internal consistency, test-retest reliability, concurrent validity, and known-group validity), sample demographic and clinical characteristics, rates of participation at baseline and week 4, and the occurrence of any loss to follow-up or missing data were examined. The response distributions for all CF-IQ items were tabulated and examined for floor and ceiling effects and sparse response categories. Note that in all analyses missing data were not imputed and only observed data were analyzed.

**Table 2:** NIVS assessments and schedule of administration.

PROM		Description	Assessment schedule	
			Baseline	Week 4
Primary variable	CF-IQ [16]	• 40-item questionnaire measures each impact concept using a 5- or 7-point verbal rating scale • Administered for psychometric validation as fit-for-purpose PROM	X	X
Validating variables	TBQ [21]	• 15-item questionnaire measuring treatment burden (i.e., taking medicine, physical activity, and impacts of treatment) • Scores range from 0–130	X	
	WPAI-SHP [22]	• 6-item questionnaire measuring the effect of a target health problem (CF) on work productivity loss and impairment of regular activities • Scores range from 0–100% (lower scores indicate reduced impairment)	X	
	PROMIS SRSF-4 [23,24]	• 4-item questionnaire assessing a person’s ability to work and participate in social and daily activities	X	
	IPQ-R [25]	• Collects a person’s perceptions of signs and symptoms of their condition(s) since the onset of illness • Collected 6 items from the Emotional domain of the IPQ-R	X	
	CFQ-R [12,26]	• Widely used PROM measuring HRQoL across several domains in people with CF, including physical, respiratory, health perception, and treatment burden • 18 items from the Physical Activity domain, Emotional domain, as well as select items from the Social Activity domain were collected	X	
	PGIS	• Single-item global scale that assesses the severity of the impact CF has on a person’s ability to do/plan things in everyday life	X	X
	PGIC	• Single-item global scale that assesses perceived change in CF limitations on a person’s ability to do/plan things		X

CF, cystic fibrosis; CF-IQ, Cystic Fibrosis Impact Questionnaire; CFQ-R, Cystic Fibrosis Questionnaire-Revised; HRQoL, health-related quality of life; IPQ-R, Illness Perception Questionnaire-Revised; NIVS, noninterventional validation study; PGIC, Patient Global Impression of Change; PGIS, Patient Global Impression of Severity; PROM, patient-reported outcome measure; PROMIS SRSF-4, Patient-Reported Outcomes Measurement Information System Social Role Short Form-4 items; TBQ, Treatment Burden Questionnaire; WPAI-SHP, Work Productivity and Activity Impairment Specific Health Problem Questionnaire.

### Part 1: Resolving item redundancy in the CF-IQ

In summary for part 1, modern psychometric method (MPM)-based local dependence statistics were employed to identify redundant item groups within domains. Any items identified as redundant were eligible for removal from the CF-IQ. Which items to retain from identified redundant item groupings were adjudicated by two qualitative focus-groups. The adjudicated solutions were evaluated in a final round of MPMs.

During qualitative development, the CF-IQ was found to consist of the following domains: Control and Burden of CF Treatment Impacts, Physical Activity Impacts, Social Activity Impacts, Emotional Impacts, and Work/School Limitation Impacts. Using MPMs (i.e., item response theory [IRT] models), the unidimensionality of each domain and any local dependence (LD) [27,28] among items within each domain of the CF-IQ were evaluated. Any items presenting with significant LD (*P* < .05) were flagged as redundant [27]. For locally dependent item combinations, items proposed for retention were those whose item content was deemed qualitatively to be most relevant to patients and had the numerically largest IRT slopes. Note that LD does not necessarily occur in pairs and in some cases multiple items were found to be jointly locally dependent.

Subsequent to redundant item removal, unidimensional IRT models were refit to each domain composed of the retained items, and model fit [29] and LD were re-evaluated [27,29]. Model fit was evaluated via the C2-based root mean squared error of approximation (RMSEA), for which a value of < 0.05 was deemed an acceptable model fit. If an acceptable model fit and no LD was observed, then that item set was deemed refined. Each domain’s refined item set proceeded to the next stage, where focus groups were asked to assess the refined item set. Two independent virtual focus groups were interviewed and observed by trained members of the research team using a semi-structured interview guide in ~90-minute sessions (S1 Appendix). Focus group members were recruited via email sent by RPV and sampled from participants who had participated in and completed the NIVS.

Participants were presented with each grouping of LD-identified redundant items, one grouping at a time, and asked which item they would retain if they could retain only one item. Participants were instructed to make their decision based on what item they found to be most relevant to their specific experience with impacts from CF. Interviews were audio recorded (with participants’ consent), transcribed verbatim, and analyzed using ATLAS.ti v8.0.

Two independent researchers coded and reviewed both focus groups’ transcripts to ensure inter-coder agreement. Coding outputs were reviewed to ensure that proposed item removal aligned with CF participant perspectives and, when needed, to resolve any inconsistencies between qualitative and psychometric evidence. When focus group data disagreed with removal decisions, the unidimensional IRT model was refit to that domain, alternating the item retained to evaluate the optimal item configuration. ‘Optimal’ was defined by the configuration associated with the best model fit and strongest IRT slope. This, along with consideration of the strength of psychometric findings, original qualitative concept elicitation, cognitive interview results, and focus group analysis, was used to arrive at the final CF-IQ item and domain composition. Please refer to the online supplement for a detailed example of the employed MPM strategy based on the physical activity impacts domain (S1 Summary).

### Part 2: Assessing reliability and validity of the refined CF-IQ

After resolving redundancy in Part 1, Part 2 involved assessing the internal consistency, test-retest reliability, concurrent validity, and known-groups validity of the CF-IQ domain scores.

The CF-IQ and validating variables collected for the NIVS are summarized in Table 2 [12,21–26]. Two global impression anchor variables were developed for this study. The Patient Global Impression of Severity (PGIS) is a self-reported, single-item, global scale subjectively assessing the severity of patients’ clinical status. Likewise, the Patient Global Impression of Change (PGIC) is a single item assessing subjective perceived change in clinical status. The FDA’s latest guidance documents, at the time of this study’s conduct, recommended using these anchor variables to support validation activities [19].

CF-IQ reliability was assessed with both internal consistency (McDonald’s *ω* and Cronbach’s α) [30,31] at baseline and test-retest reliability. Test-retest reliability was computed via the two-way, random, intraclass correlation coefficient (ICC (2,1)) [32] for CF-IQ scores between baseline and week 4 in symptomatically stable subgroups. The two symptomatically stable retest samples consisted of participants reporting no change on the PGIS between baseline and week 4 and participants reporting no change on the PGIC at week 4.

CF-IQ validity was assessed at baseline using both concurrent and known-groups validity. Concurrent validity was estimated using Pearson correlations between CF-IQ domain scores and scores from the Cystic Fibrosis Questionnaire-Revised (CFQ-R), Illness Perception Questionnaire-Revised, Patient-Reported Outcomes Measurement Information System Social Role Short Form-4 Items, PGIS, Treatment Burden Questionnaire, and Work Productivity and Activity Impairment Questionnaire-Specific Health Problem (WPAI-SHP). Known-groups validity was estimated using analysis of variance (ANOVA) models. Known anchor groups were defined on PGIS (“not limited,” “a little limited,” “moderately limited,” “severely limited,” or “extremely limited”), ppFEV_1_ groups (ppFEV_1_ ≤ median value or ppFEV_1_ > median value), and CFTRm therapy duration groups (untreated with CFTRm, treated for ≤4 weeks or treated for >4 weeks). One ANOVA was estimated for each anchor and CF-IQ domain combination. The dependent variable for each ANOVA was the CF-IQ. Known-groups contrasts were structured using reference cell coding; the reference groups for each known group were “not limited,” “ppFEV_1_ ≤ median ppFEV_1_ value,” and “untreated” in the PGIS, ppFEV_1_, and CFTRm therapy groups, respectively.

### Statistical software

MPM analyses were conducted using either the multidimensional IRT (mirt) R package [33] or flexMIRT version 3.5 [34]. Descriptive and classical test theory statistics were generated using a combination of Statistical Analysis System ([SAS] Software, version 9.4, SAS Institute Inc, Cary, NC) [35] and R statistical software (R, version 3.4.3, R Development Core Team) [36].

## Results

### Description of survey sample

**Fig 1:**
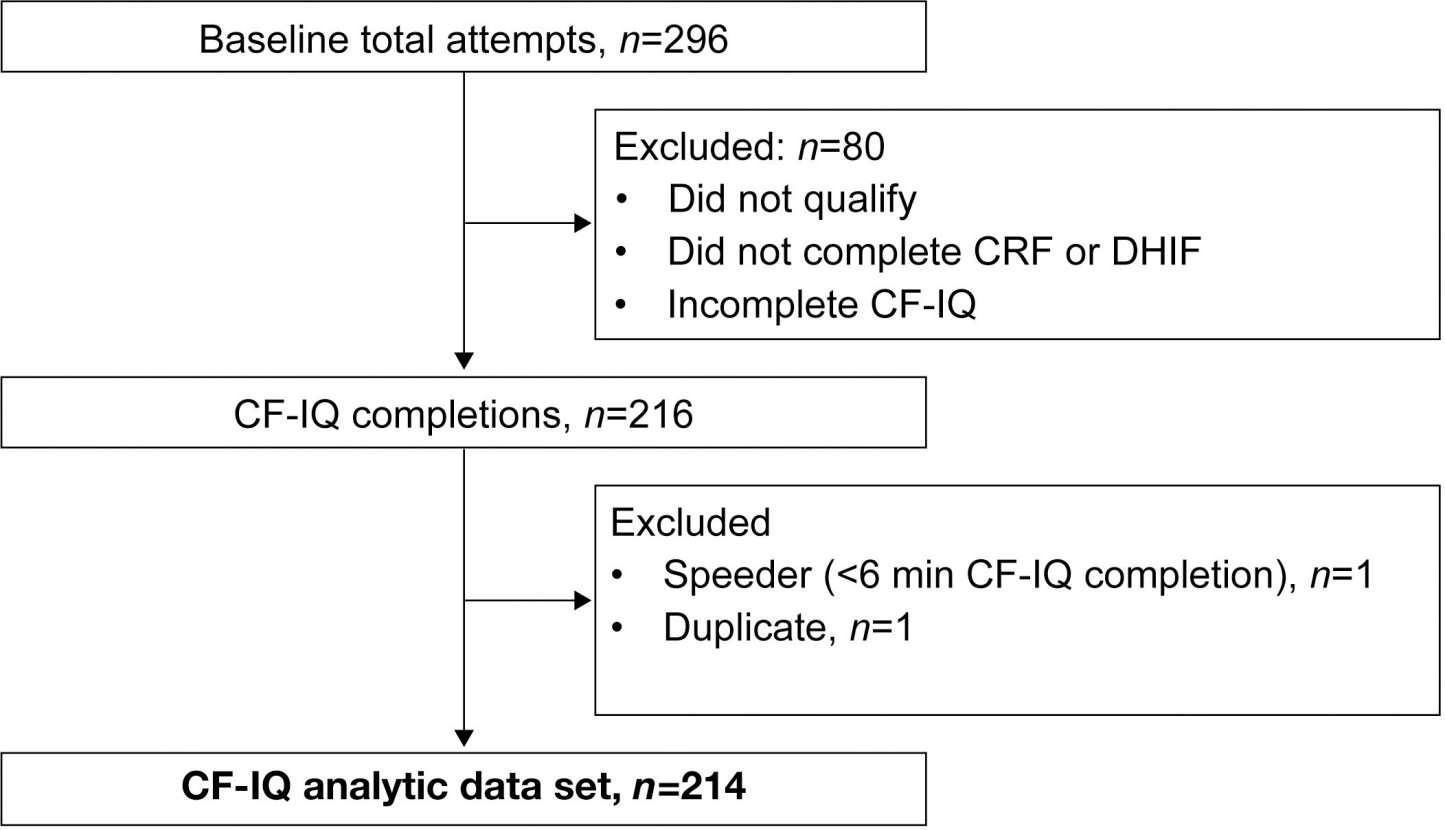
Baseline sampling flow.

At baseline, 296 RPV panelists with CF were identified and sent QuestionPro survey URLs. Of the panelists, 80 did not qualify based on eligibility criteria or did not provide complete survey data. Two of the remaining 216 panelists were removed: one for excessively fast survey completion (<6 minutes) and one for providing a duplicate record, yielding a sample of 214 at baseline, 72% response rate. The baseline sample flow is depicted in Fig 1. All 214 participants were presented with follow-up survey links at week 4 and 193 returned usable data, 90% response rate. For each CF-IQ item, a *t* test compared the average item response at baseline between participants who completed the week-4 follow-up survey and those who did not. Even without adjustment for multiplicity, no comparison was statistically significant, suggesting that attrition bias was unlikely.

### Demographics and clinical characteristics

Participants ranged in age from 18 to 72 years, with a majority (79%) being female (Table 3). Mean (standard deviation) for the most recent ppFEV_1_ was 70.0 (24.0), taken on average ≈2 months before the baseline survey. Most baseline survey participants (72.9%) experienced ≥1 pulmonary exacerbation (PEx) in the past year; 15.4% had received a lung transplant. Of 157 participants receiving CFTRm, nearly all (98.1%; n = 154) had done so for >4 weeks.

**Table 3:** Demographics and clinical characteristics of survey participants at baseline.

	Survey participants at baseline (*n* = 214)
**Demographic and Social Characteristics**	
Age, years	
Mean (SD)	33.7 (9.56)
Median (minimum, maximum)	32.0 (18.0, 72.0)
Sex, *n* (%)	
Female	169 (79.0)
Male	45 (21.0)
Racial background,^a^ *n* (%)	
American Indian or Alaska Native	3 (1.4)
Asian	3 (1.4)
Black or African American	1 (0.5)
Native Hawaiian or other Pacific Islander	0
White	211 (98.6)
Other	3 (1.4)
Marital status, *n* (%)	
Never married	97 (45.3)
Married/domestic partner	95 (44.4)
Divorced	18 (8.4)
Separated	1 (0.5)
Widowed	2 (0.9)
Other	1 (0.5)
Living/domestic situation, *n* (%)^a^	
Alone	28 (13.1)
With members of my immediate family	156 (72.9)
With other adults who are not family members	31 (14.5)
In an assisted living accommodation/residential care	0
Other	2 (0.9)
Highest level of education, *n* (%)	
Less than high school	2 (0.9)
High school diploma or equivalent	28 (13.1)
Some college but no degree	71 (33.2)
Associate’s degree	29 (13.6)
Bachelor’s degree	62 (29.0)
Master’s degree	18 (8.4)
Doctoral or professional degree	4 (1.9)
Employment status, *n* (%)^a^	
Student	30 (14.0)
Employed for wages	95 (44.4)
Homemaker	18 (8.4)
Retired	2 (0.9)
Unemployed	11 (5.1)
Disabled (or on disability or leave of absence)	97 (45.3)
Other	1 (0.5)
Amount of work per week performed by participants who are employed for wages, hours	
Mean (SD)	33.2 (12.0)
Median (minimum, maximum)	38.0 (3.00, 70.0)
Yearly household income, *n* (%)	
Under $9999	24 (11.2)
$10,000 to $14,999	31 (14.5)
$15,000 to $24,999	19 (8.9)
$25,000 to $34,999	20 (9.3)
$35,000 to $49,999	26 (12.1)
$50,000 to $74,999	35 (16.4)
$75,000 to $99,999	21 (9.8)
$100,000 to $124,999	15 (7.0)
$125,000 and over	23 (10.7)
**Physical and Clinical Characteristics**	
Height, mean (SD), cm	162.8 (8.59)
Weight, mean (SD), kg	63.0 (14.5)
Most recent self-reported ppFEV_1_, mean (SD)	70.0 (24.0)
Time since last ppFEV_1_ reading, mean (SD), months	1.76 (1.32)
Experienced ≥1 PEx in the past year, *n* (%)	156 (72.9)
Number of PEx in the past year in participants who experienced ≥1 PEx in the past year^b^^,^^c^	
Mean (SD)	3.35 (6.51)
Median (minimum, maximum)	2.00 (1.00, 80.0)
Hospitalized or received IV antibiotics for PEx in the past year, *n* (%)^c^	
No	32 (20.5)
Yes; received IV antibiotics but was not hospitalized	28 (18.0)
Yes; hospitalized but did not receive IV antibiotics	0
Yes; hospitalized and received IV antibiotics	96 (61.5)
If hospitalized for PEx, time since discharge, weeks^c^	
Mean (SD)	17.8 (13.9)
Median (minimum, maximum)	16.0 (0, 52.0)
Current therapies for CF, *n* (%)^a^	
CFTRm	157 (73.4)
Mucolytics	162 (75.7)
Bronchodilators	172 (80.4)
Digestive enzymes	186 (86.9)
Anti-inflammatory therapy	56 (26.2)
Antibiotics	157 (73.4)
Other therapy	17 (7.9)
No current therapy for CF	1 (0.5)
Duration of current CFTRm use, *n* (%)^d^	
<4 weeks	12 (7.6)
≥4 weeks	145 (92.4)
Duration of any CFTRm use, *n* (%)^d^	
<4 weeks	3 (1.9)
≥4 weeks	154 (98.1)
Frequency of CF care team check-ups, *n* (%)	
Monthly	26 (12.1)
Quarterly	157 (73.4)
Twice a year	12 (5.6)
Yearly	5 (2.3)
Other	14 (6.5)
Received lung transplant, *n* (%)	33 (15.4)
Uses airway clearance techniques, *n* (%)	170 (79.4)
Uses vascular assist devices, *n* (%)	
Yes	7 (3.3)
No	182 (85.0)
I don’t know	25 (11.7)

CF, cystic fibrosis; CF-IQ, Cystic Fibrosis Impact Questionnaire; CFTR, cystic fibrosis transmembrane conductance regulator; CFTRm, cystic fibrosis transmembrane conductance regulator modulator; PEx, pulmonary exacerbation; IV, intravenous; ppFEV_1_, percent predicted forced expiratory volume in 1 second.

^a^ The total percentage exceeds 100 because participants were able to select >1 option.

^b^ The mean number of exacerbations in the full CF-IQ sample at baseline is biased by a large outlier (80 exacerbations at baseline). The median better reflects the data.

^c^ Calculated in participants who experienced PEx in the past year (*n* = 156).

^d^ Calculated in participants who were receiving a CFTRm (*n* = 157).

### Descriptive assessment of CF-IQ

Almost all CF-IQ items demonstrated some response category sparseness (categories with 10% or less of the sample endorsing); however, this was not a barrier to analysis because sufficient representation was observed across response categories for all CF-IQ items. Overall, most impacts were mildly to moderately severe, with the middle three response categories heavily endorsed.

### Part 1: Resolving item redundancy in the CF-IQ

Across the five prespecified domains, 31 (78% of 40 CF-IQ items) CF-IQ items presented with substantial LD across 13 patterns described in Table 4. Observed patterns of LD involved a minimum of two to a maximum of four items. Of these 31 items, 17 (43% of the original 40 CF-IQ items) were found to be statistical drivers of the 13 unique LD patterns. These 17 items were identified as redundant and therefore candidates for removal from the CF-IQ. For example, in the Physical Activity domain, the following items presented with significant LD: CF-IQ item 2 “How difficult was it for you to keep up with others while running” and CF-IQ item 6 “How much of the time did you need to take breaks or rest in order to complete tasks at home, work, or school?”; while both had strong slopes (2.33 and 2.86, respectively), CF-IQ item 6 had the higher slope and was therefore retained. In circumstances where multiple items were found to be jointly locally dependent, the smallest set of items that could be removed and result in LD resolution were identified. For example, in the case of the Emotional Domain items 15,16,17, and 19, with item content of “feeling frustrated”, “feeling irritable”, “feeling moody” and “feeling stressed”, respectively, were found to be locally dependent and the local dependence was resolved by retaining item 15, “feeling frustrated”. When these 17 items were removed and the unidimensional IRT models refit to each domain, model fit was perfect (RMSEA = 0.00) for each domain.

**Table 4:** Summary of item removal findings.

			Determining which item to retain		
Domain	Unique LD Grouping	Redundant items	Based on initial psychometrics	Based on focus group^a^	Based on totality of evidence^b^
Control and Burden of CF Treatment	1	33, 35	35	33	33
	2	34, 36	36	34	36
	3	39, 40	40	40	40
Physical Activity	4	3, 4	4	4	4
	5	5, 26	5	5	5
	6	2, 6, 25, 29	6	6	6
Social Activity	7	22, 24	22	22	22
	8	23, 35	23	23	23
	9	30, 31, 32	31	31	31
Emotional	10	11, 14	11	11	11
	11	15, 16, 17, 19	15	15	15
	12	18, 20	18	18	18
Work/School Limitations	13	37, 38	38	38	38

^a^ Items retained after review of psychometric, original concept elicitation and cognitive interviews, and focus group evidence.

^b^ Final agreed-upon decision achieved among study investigators.

The two independent focus groups were presented the 31 items defining the 13 unique LD patterns and asked to decide which items were redundant and which to retain. The focus groups agreed that 16 of the 17 (94%) items deemed redundant by psychometric evidence, were in fact redundant and should be removed. The one disagreement with the psychometric evidence involved the first unique LD grouping given in Table 4. Both focus groups agreed that item 33 (“Bothered by the amount of time needed to complete CF treatments?”) would evaluate the overall time spent on CF treatment; and that this would provide a more holistic view of treatment burden compared to that of item 35 (“Frustrated about activities you missed because of the time you spent on CF treatment?”), which focused on frustration with activities missed due to CF treatment. With respect to unique LD grouping 2, focus groups (FG) 1 and 2 disagreed on which item to retain: FG 1 agreed with the psychometric evidence and FG2 did not. To resolve which item in each item pair (LD grouping 1: 33 & 35, and LD grouping 2: 34 &36) should be retained, IRT models were refit to the control and burden of treatment domain; and following the criteria described in methods, items 33 and 36 were retained. Therefore, the final item content for the CF-IQ was in agreement with the feedback received from participants in two focus groups.

The final C2-root mean square error approximation (RMSEA)-based model fit and *ω*-based internal consistency estimates for each of the five CF-IQ domains, defined upon the final 23 CF-IQ items (40 minus 17 items), are presented in Table 5. Per the proofs given by McDonald *et al*. α underestimates internal consistency and is, therefore, negatively biased relative to the ω. ω is therefore optimal at the stage of domain evaluation [34]. All domains for which model fit was estimable (all but Work/School Limitations) exhibited perfect fit (C2-based RMSEA point estimates of zero and null *p* values) and were associated with superlative internal consistency, with estimates of *ω* ranging between 0.87 and 0.92. The domain modifications and IRT item slopes corresponding to the final model specification are given in Table 6. As implied by the magnitude of the observed *ω*-based internal consistency, IRT slopes for the retained items were uniformly robust, with all exceeding 1.0 and some approaching deterministic slope magnitudes (e.g., CF-IQ item 22 “Difficulty participating in activities you enjoy doing” and item 28 “Hard time keeping up with daily tasks at home/work/school”). Items not retained in the final model are illustrated in grey text in Table 6.

**Table 5:** Final C2-RMSEA-based model fit and Ω-based internal consistency estimates for final CF-IQ item pool per domain.

Domain	RMSEA	*p* value	*ω*
Physical Activity	0.0	0.460	0.92
Control and Burden of CF Treatment	0.0	0.600	0.88
Social Activity	0.0	0.579	0.92
Emotional	0.0	0.854	0.90
Work/School Limitations	NA^a^	NA^a^	0.87

CF, cystic fibrosis; CF-IQ, Cystic Fibrosis Impact Questionnaire; NA, not applicable; RMSEA, root mean square error approximation.

^a^ The Work/School Limitations domain, with only 3 final items, lacked the necessary degrees of freedom to calculate these values.

**Table 6:** Summary of descriptive assessment of CF-IQ items (unretained items shown in gray).

Domain	Item no.	CF-IQ item content	Items retained after psychometric and focus group evaluation	
			Model slope^a^	Retained in final model
Control and Burden of CF Treatment Impacts	8	Worried about getting sick?	2.20	Yes
	9	Feel confident in your ability to manage CF?	1.91	Yes
	10	Frequently feel your health was out of your control?	2.65	Yes
	33^b^	Bothered by the amount of time needed to complete CF treatments?	1.33	Yes
	34	Amount of time spent on extra CF treatments that were not a part of your usual routine?	NA	No
	35^c^	Frustrated about activities you missed because of the time you spent on CF treatment?	NA	No
	36	Worried about the possibility of having to be hospitalized?	2.37	Yes
	39	Feel that you are as healthy as others your age?	NA	No
	40	Feel confident that your health will allow you to do the things you want to in the future?	1.28	Yes
Physical Activity Impacts	1	Difficulty keeping up with others while walking?	3.01	Yes
	2	Difficulty keeping up with others while running?	NA	No
	3	Difficulty climbing stairs?	NA	No
	4	Difficulty doing sports/exercise?	2.42	Yes
	5	Difficulty doing housework/yardwork or run errands?	3.67	Yes
	6^c^	Amount of time needed to take breaks or rest to complete everyday tasks?	3.01	Yes
	7	Feel physically strong?	1.70	Yes
	25	Feel too sick to attend work/school?	NA	No
	26	Coughing wake you up at night?	NA	No
	27	Get less sleep than needed?	1.47	Yes
	28^c^	Hard time keeping up with daily tasks at home/work/school?	3.84	Yes
	29	Repeatedly needed extra help from others?	NA	No
Social Activity Impacts	21	Skip social opportunities?	3.36	Yes
	22	Difficulty participating in activities you enjoy doing?	5.00	Yes
	23	Feel isolated from others?	2.95	Yes
	24	Your CF symptoms cause you to change plans you already made?	NA	No
	30	CF made your relationships with friends difficult?	NA	No
	31	CF made your relationships with family members difficult?	2.04	Yes
	32	CF interfered with the way your family gets along?	NA	No
	35^d^	Feel frustrated about activities you missed because of the time spent on CF treatment?	NA	No
Emotional Impacts	11	Feel angry?	3.01	Yes
	12	Feel anxious?	2.35	Yes
	13	Feel embarrassed?	1.30	Yes
	14	Feel fearful?	NA	No
	15	Feel frustrated?	2.24	Yes
	16	Feel irritable?	NA	No
	17	Feel moody?	NA	No
	18	Feel sad?	3.09	Yes
	19	Feel stressed?	NA	No
	20	Feel depressed?	NA	No
Work/School Limitation Impacts	6^c^	Needed time to take breaks or rest to complete tasks?	3.12	Yes
	28^c^	Hard time keeping up with daily tasks at home/work/school?	4.66	Yes
	37	Feel limited about your job or career choices?	NA	No
	38	Feel limited about the goals you can set for your life?	1.50	Yes

CF, cystic fibrosis; CF-IQ, Cystic Fibrosis Impact Questionnaire; IRT, item response theory; NA, not applicable.

^a^ Models were refit after all item reductions to yield the final model slopes given here.

^b^ Focus group evaluation of redundancy resulted in only 1 disagreement with psychometric evidence. Psychometric evidence supported retaining item 35 but focus group evidence supported retaining item 33. When the IRT model for the Control and Burden of CF Treatment Impacts domain was refit, retaining item 33 in place of item 35, the model fit and psychometric properties remained acceptably robust, and item 33 was retained in place of item 35.

^c^ Items 6, 28, and 35 were each initially included in 2 separate domains. Items 6 and 28 were each kept in both domains that they were included in. Accordingly, these items each have 2 different model slopes reported: 1 for each domain in which they were included. Item 35 was dropped from both domains in which it was included.

Combined, this evidence concluded the item finalization process. CF-IQ domain scoring was based on unit-weighted sum scores as supported by the *ω* statistics [28,37], under which higher scores indicate greater disease impact. The refined CF-IQ is available from the Mapi Research Trust at: https://eprovide.mapi-trust.org/instruments/cystic-fibrosis-impact-questionnaire.

### Part 2: Assessing reliability and validity of the refined CF-IQ

**Fig 2:**
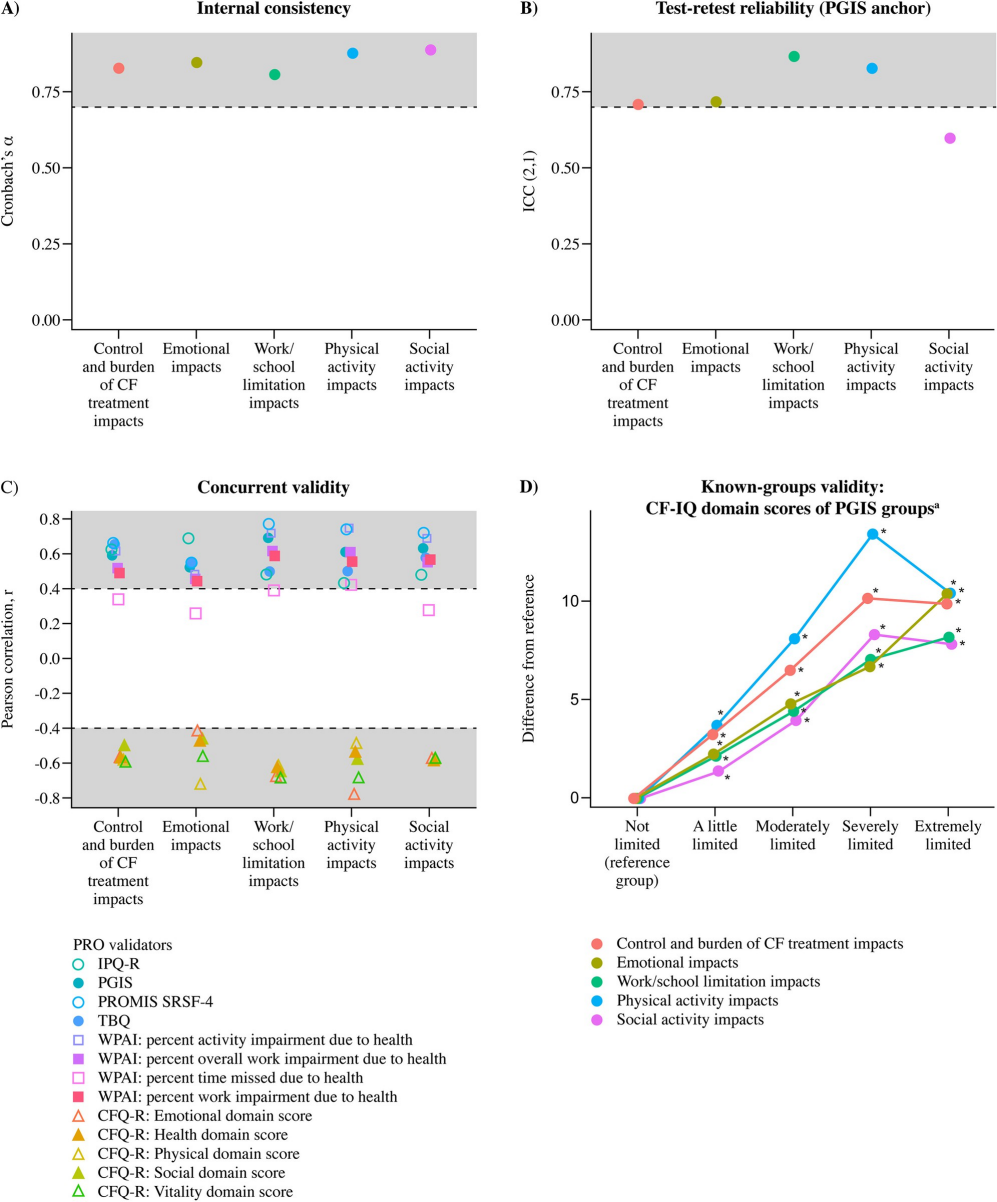
Classical methods for CF-IQ scores: Reliability and validity. Plots of the (A) internal consistency, (B) test-retest reliability, (C) concurrent validity, and (D) known-groups validity of the CF-IQ domains CF-IQ, Cystic Fibrosis Impact Questionnaire.

Reliability and validity for the five domains defined on the final 23-item CF-IQ were robust (Fig 2A–2D). All CF-IQ domains had acceptable internal consistency, as demonstrated by Cronbach’s α values ≥ 0.7. As shown in Fig 2A, Cronbach’s α values ranged from 0.81 to 0.89, with the Physical Activity Impacts and Social Activity Impacts domains having the highest estimates; the Work/School Limitation Impacts domain had the lowest estimate.

The Control and Burden of CF Treatment Impacts, Emotional Impacts, Work/School Limitation Impacts, and Physical Activity Impacts domains demonstrated acceptable test-retest reliability for the PGIS anchor, as indicated by ICC values ≥ 0.7 (Fig 2B). The Control and Burden of CF Treatment Impacts, Emotional Impacts, and Social Activity Impacts domains did not reach acceptable test-retest reliability for the PGIC anchor (data not shown).

Concurrent validity was robust, with 96% of estimated correlations between CF-IQ domains and validators meeting or exceeding the criterion of |r| ≥ 0.4, indicating that those instruments expected to measure similar constructs to the CF-IQ domains do appear to measure similar constructs (Fig 2C). Three correlations between the CF-IQ and the WPAI-SHP validator failed to meet the criterion for concurrent validity. Note that Fig 2C presents correlation magnitudes graphically relative to the prespecified cut-offs defining acceptable concurrent validity (|r| ≥ 0.4). While not all numerical values are clearly observable (e.g., when one validator is correlated at 0.48 and another correlated at 0.50, proximity will result in graphical overlap), there are five discriminant validators (the CFQ-R domains as they are reverse-scored relative to CF-IQ) and eight convergent validators. Therefore, readers can evaluate the relative distance of correlations to thresholds and each other, knowing there will be five discriminant correlations for each domain in proximity to -0.4 and eight convergent correlations for each domain in proximity to 0.4.

Known-groups validity evidence estimated in PGIS known groups was strong, though more modest when estimated across ppFEV_1_ and CFTRm known groups (S1 Table). For all CF-IQ domains, each severity group based on the PGIS had domain scores that were significantly different from the reference group (Fig 2D). In each domain, the effect group score means were higher than the reference group score mean, and the semipartial ω^2^ estimate of the effect size of the PGIS group predictor exceeded the prespecified criterion of 0.05 (semipartial ω^2^ range: 0.25–0.47 across domains). This suggests that groups expected to have CF-IQ scores indicating more severe disease do show this trend.

## Discussion

The CF-IQ was developed as a CF-specific PROM following current PROM development guidelines [17,18] and standard qualitative practice [16]. In this study, a novel, hybrid study design was utilized to increase patient-centricity to resolve item redundancy and validate the CF-IQ. This hybrid approach allowed the collection of a large validation sample. The evidence derived from this large validation sample was then refined with the assistance of patient input obtained from patient focus group discussions. A total of 17 items were identified as redundant and removed.

Based on psychometric evidence, focus group interviews confirmed all but one item removal decision. This discrepancy between the psychometric evidence and focus group findings was not concerning, as both items in the redundant pair exhibited similar strong item performance; the instrument’s overall performance was excellent with either retained. The hybrid design and resulting evidence demonstrated strengths of linking psychometric and qualitative evidence to clarify guidance from the patient voice. This study illustrates how evidence obtained from correctly implemented MPMs can corroborate the patient perspective and vice versa.

After item pool finalization, 23 of 40 items were retained across five domains: Control and Burden of CF Treatment Impacts, Physical Activity Impacts, Social Activity Impacts, Emotional Impacts, and Work/School Limitation Impacts. Classical validation (reliability and validity) of the 23-item CF-IQ demonstrated that it is a psychometrically robust PROM for use among adults with CF and is fit for purpose in providing a holistic understanding of the impact of CF on QoL. However, results were not uniform. For test-retest reliability, three domains did not meet the prespecified criterion for the PGIC, while the majority of the domains demonstrated acceptable test-retest reliability for the PGIS anchor. Estimates conditioned on the PGIS generally outperform those conditioned on the PGIC, when used for validation. This is routinely explained as a function of recall bias. The PGIC requires the individual to evaluate change over time and is expected to carry recall bias; this attenuates the ICC relative to the PGIS, an absolute measure of perceived severity at the time of survey completion. Validation also confirmed that the CF-IQ domain scores had a high level of agreement with other validated PROMs, including the widely used CFQ-R. The strong correlation observed in concurrent validity between CF-IQ and other validating PROMs allowed us to confirm that CF-IQ accurately measured overlapping concepts across validated PROMs. Note that a relevant distinction between the CFQ-R and CF-IQ is that the latter was developed to characterize impacts reflective of the longer lifespans prevalent in the CFTRm era. To this point, the CF-IQ item responses reflected moderate to severe impacts with polar categories under-endorsed. This is in substantial contrast to patterns observed in the pre-CFTRm era, under which the vast majority of legacy PROM-based impact items possessed non-trivial ceiling effects [26].

This study was conducted during the early months of the SARs-CoV-2 outbreak in January through April 2020. This study was designed (prior to SARs-CoV-2) to employ sampling via a virtual collection deployed in RPV’s CF panel to facilitate efficient data collection. As a result, in-person site-based sampling and laboratory-based confirmation of inclusion/exclusion criteria were infeasible. However, the virtual design allowed us to complete data collection efficiently and unaffected by SARs-CoV-2. The QuestionPro survey environment enabled collection of high-integrity data while preserving methodologic principles that ensured data rigor within a HIPAA-compliant framework. This approach allowed us to achieve an adequate representative sample for validation efforts supporting continued research. Response rates were high (90% at follow-up) and statistical testing provided further evidence against sampling bias in the survey collection.

An additional advantage conferred by the virtual survey sampling design was rapid access to a large sample size in a rare disease population; however, virtual survey design has inherent limitations. Survey respondents were self-selected into the panel, so they may not represent the wider CF population. Consistent with another large online survey of people with CF [38], women were over-represented in the sample; however, lack of evidence of differential item functioning by sex indicates that responses were unbiased. Clinical information (e.g., lung function) was self-reported by study participants and accuracy of these values could not be verified; however, based on the reported data, the study sample appears to be generally similar to the adult population in the US Cystic Fibrosis Foundation Patient Registry [6]. The study did not collect information on CF genotype in participants; therefore, potential response differences by genotype or treatment availability could not be explored. Finally, it is possible that respondents intentionally entered incorrect or false responses; this was mitigated by excluding a small number of participants with excessively fast survey completion or duplicate records.

In conclusion, the CF-IQ is a psychometrically robust PROM capturing patient-centric outcomes to understand the broad life impacts of CF and potential benefits of novel treatments in an adult population with CF. These findings support the further development of the CF-IQ alongside clinical endpoints in future interventional trials and studies with expanded populations. Assessments of the responsiveness of the CF-IQ to change and clinical significance thresholds will be addressed in an ongoing interventional trial.

## Supporting information

S1 Appendix Additional information related to the focus group interviews including interview guide. (DOCX)

S1 Summary Illustrative example of modern psychometric methods. (DOCX)

S1 Table Known-groups validity at baseline across ppFEV_1_ and CFTRm known groups. (DOCX)
