# Supplementary material for: Psychometric validation of the Cystic Fibrosis Impact Questionnaire (CF-IQ): A patient-reported outcome assessing impacts of cystic fibrosis
Source: PLoS One. 2025 Jan 24;20(1):e0317775. doi: 10.1371/journal.pone.0317775 (PMC11761112; doi:10.1371/journal.pone.0317775)
Supplement: S1 Appendix — (DOCX) [file pone.0317775.s001.docx]

**S1 Appendix: Additional information related to the focus group interviews including interview guide**

1. **Research team and reflexivity**

| **Interviewer/Facilitator/Researcher Characteristics** | | | | | **Relationship with participants** | |
| --- | --- | --- | --- | --- | --- | --- |
| **Name** | **Qualifications** | **Occupation at time of study** | **Gender** | **What experience or training did the researcher have?** | **Was a relationship established prior to study commencement?** | **What did the participants know about the researcher?** |
| Maya Gerstein | PhD | Associate Director | Female | 12+ years of experience conducting qualitative interviews | No relationship outside of a professional one. | Participants were only aware of the researchers’ names, but were well-informed about the research's purpose, its stakeholders, data privacy protocols, and reimbursement terms for participating in interviews. This comprehensive communication strategy demonstrated a commitment to transparency, ethical practice, and respect for participants' involvement in the research process. |
| Alyssa Uzumcu | BS | Senior Research Analyst | Female | 5+ years of experience conducting qualitative interviews |  |  |

1. **Interviews**

Insights from the initial wave of interviews served as a quasi-pilot testing function. During these initial interviews, researchers would observe interviewees' reactions and thoughts, specifically focusing on their agreement or disagreement with items retained or removed based on their overall experiences with a particular symptom. The feedback would be used reflexively to adjust the questions as needed for the next round of interviews.

Besides the participants and researchers, no one else was present at the time of the interviews. No repeat interviews were carried out. Observer-reported focus group notes were completed as part of the data collection process and were designed to capture various non-verbal cues expressed during the focus group sessions. Transcripts were not returned to participants for comment and/or correction.

1. **Focus group interview guide**

| **Section** | **Objective** | **Estimated Time (min)** |
| --- | --- | --- |
| 1. Introduction | To provide an overview of how the focus group will be conducted, reassure confidentiality, answer any questions before beginning the focus group | 5 |
| 1. CF-IQ | To understand if the questions reduced or retained from the CF-IQ reflect participants’ experience with CF. Domains reviewed include Control and Burden of CF, Physical Activity, Social Activity, Emotional, and Work/School Limitations | 60 |
| 1. Closing Remarks | To capture any final comments and thank the participants for his/her time | 5 |
| **Total Time** |  | **~80-90** |

**SECTION 1: Introduction**

***Time: ~5 minutes***

| **Objective:** To provide an overview of how the interview will be conducted, reassure confidentiality, answer any questions before beginning interview |
| --- |

**Introduce Yourself**

- My name is ___(name)___. I work for Pharmerit International, a health research organization that conducts studies about how patients experience health conditions and/or their treatments. Thank you for agreeing to participate in this research.

**Discuss the purpose of the interview**

- You were invited to today’s focus group because you and the other **__**(*insert number*)**__** participants with CF in this focus group session previously completed two questionnaires that asked you about the symptoms you experience with CF and the impact that CF has had on your life. Since then, the research team has proposed some changes to one of those questionnaires. The purpose of today’s focus group is to understand if those changes still reflect your experience with Cystic Fibrosis. We are also interested in your perspective on the symptom questionnaire you completed and will ask you some questions about your interpretation of the questions that are part of that survey.
- As a reminder, no drug or any other treatment will be given as part of the study.
- Today’s focus group will take approximately 90 minutes. During that time, I will be asking you questions about your view of questions included in the questionnaires, covering symptoms, impacts, and treatment priorities.
- Please be open in our discussion today and share whatever is on your mind. There are no right and wrong answers. I am interested in hearing your thoughts and perspectives on the questionnaires given your experience with Cystic Fibrosis.

**Rules of Engagement**

- There are no right or wrong answers to any of the questions I am going to ask. Your response may reflect your opinion or impression based on your personal experience with Cystic Fibrosis. All experiences are important and valid. Not everyone is going to agree with what you say and that is okay. People have different opinions and experiences and we want to hear all of the different perspectives and thoughts that each of you have.
- I want to hear from everyone:
  - If we have not heard from you, I may ask you for your ideas
  - I want to make sure everyone has an opportunity to talk about each topic, so there may be times when I need to move on to another person to let them contribute
  - If we get off topic, I may need to interrupt you to bring us back on track
- Please refrain from any outside distractions during our conversation. It is distracting to others and it will make it very difficult for us to follow the conversation on the audio recording.
  - Please feel free to step out as you need to. We just ask that you do so with as little interruption to the discussion as possible and return as quickly as you can so we can continue to capture your perspective in this discussion.
- We respect the privacy of this group and will keep our discussion confidential and private, and we ask that you respect the individuals in the group and do the same
  - We will call each other only by first names or the names you have entered into the Microsoft Teams environment
  - We will not collect any identifying information from you

**Remind patient of recording**

- I want to remind you that this interview will be recorded. If a name is used in the recording, it will be removed from the transcript prior to any study analysis.
- I will only be using the audio-recording of this focus group to remind me of the important things you share and to make sure that I have accurately captured what you say. This also helps me give my full attention to you and the group during the focus group. The recording will be transcribed. The transcript from your focus group, along with the transcripts from other focus groups, will help us to capture the experience of people living with CF and their view on the questionnaires we talk about today.
- Please try to speak clearly and loud enough so that you can be heard on the recording.

**Reassure participant of confidentiality**

- I want to remind you that your name and contact information will remain confidential and only with the staff directly involved with this study. Any information that you provide will be reported in a way that protects your privacy by avoiding any mention of any information that could identify you individually.
- Do you have any questions before we start?
- I will now start the audio-recording.

***INTERVIEWER: START recording interview now.***

🗣 *Today is ___(date)___. I am speaking with focus group ___(ID)___ for project number 19143.*

**SECTION 2: CF-IQ**

***Time: ~60 minutes***

**Objective:** To understand participants’ view on retained or removed CF-IQ questions based on their experience with Cystic Fibrosis in relation to the instrument’s validation analysis.

*To begin, we will review each section of the CF-IQ. The Cystic Fibrosis Impacts Questionnaire (CF-IQ) is a questionnaire designed to measure the impact and disease burden of Cystic Fibrosis (CF) on your everyday life.*

*As we go through each section, we will review certain questions that were considered similar or alike. In these cases, one question was dropped, and one question was kept based on patterns of survey responses. The purpose of removing questions from this survey is to reduce the redundancy and minimize the time it takes to respond to the CF-IQ, making it easier for individuals with CF to complete it. When we review each similar question pair (or group), I will ask you to provide feedback on whether you agree with the question that was kept or dropped based on your experience with CF and why.*

*We may not discuss questions in the order they appear in the CF-IQ questionnaire, so please focus more on the content of each question rather than the numbers attributed to each question.*

**Control and Burden of CF Treatment Domain (Slides 2 – 6)**

*The first section of the CF-IQ we will look at asks questions related to the Burden of CF Treatment on your life. Questions in this section cover treatments, hospitalizations, care management, and health outcomes.*

1. **The first similar pair is question 33 and question 35.** *Interviewer read questions out loud.* **These two questions were meant to capture the burden of CF treatments on your daily life.** **Would you respond to these similarly? Why or why not?**
   - *PROBE: If respondent needs clarity*: On a 1-5 scale, with 1 being not at all and 5 being extremely bothered/frustrated, would you rate these similarly? Why or why not?
   - PROBE: In your view, what does “bother” mean? Is that the same or different from “frustrated” in your experience? Why?
   - PROBE: Both questions reflect the burden of CF treatments on your daily life. Does one questions better reflect your personal experience more than the other? How so?

*Interview instruction: Highlight the retained question in the slide to show the participant*

- - PROBE: Ultimately, we ended up keeping question 35 because responses from survey participants like yourself seemed to suggest that feeling frustrated by the activities you missed because of CF treatment is a better description of treatment burden than how bothered someone is by the amount of time needed to complete their CF treatments. What is your reaction to this? Do you agree with this decision?

1. **The second similar pair is questions 34 and 36.** *Interviewer read questions out loud.* **These two questions are intended to capture information about extra care or treatments that are not part of your usual CF treatment. Would you respond to these similarly? Why or why not?**
   - *PROBE: If respondent needs clarity:* On a 1-5 scale, with 1 being not at all and 5 being extremely worried or an extreme amount, would you rate these similarly? Why or why not?

- PROBE: Both of these questions speak to parts of your CF treatment that may be outside the context of your usual care. Is one of these questions more relevant to your CF experience than the other?

*Interview instruction: Highlight the retained question in the slide to show the participant*

- PROBE: Ultimately, we ended up keeping question 36 because responses from survey participants like yourself seemed to suggest that worrying about hospitalization is a better way to describe treatment burden than time spent on extra CF treatments. What is your reaction to this? Do you agree with this decision?

1. **The last similar pair in this section is questions 39 and 40.** *Interviewer read questions out loud.* **Both of these questions are intended to capture how you perceive the status of your overall health. Would you respond to these similarly? Why or why not?**
   - - *PROBE: If respondent needs clarity*: On a 1-5 scale, with 1 being not at all and 5 being extremely or extremely confident, would you rate these similarly?
   - PROBE: How do you think about your health? Do you think about your health in comparison to your peers or in terms what your future may look like?
   - PROBE: Both of these questions speak to how you perceive the status of your personal health. Does one of these questions better capture this?

*Interview instruction: Highlight the retained question in the slide to show the participant*

- PROBE: Ultimately, we ended up keeping question 40 because responses from survey participants like yourself questions seemed to suggest that confidence in future health was a more relevant description of overall health than comparing health to their peers. What is your reaction to this? Do you agree with this decision?

1. **As we review the final list of questions in this section, both the original and revised question list, we are able to see how many questions have been removed and kept. Some of the reasons we drop questions from these kinds of questionnaires is to remove redundancy and to reduce the time-burden on people who are taking it. In this section, we kept 6 of the 9 questions. Is this a meaningful reduction in your mind?**
   - PROBE: Is reducing the time to complete this section worth it to you given the things we may not capture about your CF experience?
   - PROBE: Are there any questions that were removed that you think should be kept?

**Physical Activity Domain (Slides 7 – 13)**

*The next section we will review focuses on the impact of CF on your physical activity. Questions in this section ranged from impacts on your sleep to the daily and physical activity limitations you experience due to your CF.*

1. **The first similar pair is questions 5 and 26.** *Interviewer read questions out loud.* **On the whole, individuals responded to these questions similarly. This may be because lack of sleep due to coughing impacts your ability to do everyday activities. Is that consistent with your experience?**
   - PROBE: Would you respond to these similarly? Why or why not?
     - *PROBE: If respondent needs clarity*: On a 1-5 scale, with 1 being not at all and 5 being always or extremely difficult, would you rate these similarly?

*Interview instruction: Highlight the retained question in the slide to show the participant*

- PROBE: Ultimately, we decided to keep question 5 because responses from survey participants like yourself seemed to suggest that a person’s ability to participate in everyday activities is a more relevant way to describe physical limitations than how often cough kept someone up at night. There is also another question on sleep included in the questionnaire, so we felt that this topic had been covered. What is your reaction to this? Do you agree with this decision?

1. **The second similar pair is question 3 and 4.** *Interviewer read questions out loud.* **These two questions reflect the difficulty you may experience participating in physical activities as a result of your CF. Would you respond to these similarly? Why or why not?**
   - - *PROBE: If respondent needs clarity*: On a 1-5 scale, with 1 being not at all and 5 being extremely difficult, would you rate these similarly?

- PROBE: Do you consider climbing stairs to be a form of a physically demanding activities? Why or why not?

*Interview instruction: Highlight the retained question in the slide to show the participant*

- PROBE: Ultimately, we ended up keeping question 4 because responses from survey participants like yourself suggested that difficulty participating in physically demanding activities was a better description of physical limitations than difficulty climbing stairs. What is your reaction to this? Do you agree with this decision?

1. **The last question grouping in this section includes questions 2, 6, 25, and 29.** *Interviewer read questions out loud* **These questions were designed to capture physical limitations due to your CF. Of these four questions, which one best reflects your personal experience with CF? Why?**

- PROBE: These questions speak to the physical limitations you may experience as a result of your CF. Would you respond to these items similarly? How so?
  - - *PROBE: If respondent needs clarity*: On a 1-5 scale, with 1 being not at all and 5 being extremely difficult, would you rate these similarly?

*Interview instruction: Highlight the retained question in the slide to show the participant*

- PROBE: Ultimately, we ended up keeping question 6 because responses from survey participants like yourself suggested that taking breaks or rest from activities captures the other physical limitations that are described in the three questions we dropped (difficulty running, feeling too sick to attend work or school, or needing extra help from others). What is your reaction to this? Do you agree with this decision?

1. **As we review the final list of questions in this section, both the original and revised question list, we are able to see how many questions have been removed and kept. Again, some of the reasons we drop questions from these kinds of questionnaires is to remove redundancy and to reduce the time-burden on people who are taking it. In this section, we kept 7 of the 12 questions. Is this a meaningful reduction in your mind?**
   - PROBE: Is reducing the time to complete this section worth it to you given the things we may not capture about your CF experience?
   - PROBE: Are there any questions that were removed that you feel should be kept?

**Social Activity Domain (Slides 14 – 18)**

*In this next section, we will review questions related to the impact of CF on your Social Activities. The questions in this section cover plans and interactions with friends and family as well as any disruptions to leisure activities due to your CF.*

1. **The first similar pair is questions 22 and 24.** *Interviewer read questions out loud.* **These questions are intended to capture disruptions to social activities that you participate in. Would you respond to these similarly? Why or why not?**
   - - *PROBE: If respondent needs clarity*: On a 1-5 scale, with 1 being not at all and 5 being extremely difficult or always, would you rate these similarly?

- PROBE: Both questions reflect challenges you may have encountered engaging in social activities due to your CF. Does one question reflect your personal experience more than the other? How so?

*Interview instruction: Highlight the retained question in the slide to show the participant*

- PROBE: Ultimately, we ended up keeping question 22 because responses from survey participants like yourself suggested that difficulty participating in social activities was a more relevant way to describe social limitations than changing plans due to CF symptoms. What is your reaction to this? Do you agree with this decision?

1. **The second similar pair is questions 23 and 35.** *Interviewer read questions out loud* **These two questions were designed to capture any impacts CF has had on your ability to participate in social activities. Would you respond to these similarly? Why or why not?**
   - - *PROBE: If respondent needs clarity*: On a 1-5 scale, with 1 being not at all and 5 being extremely or always, would you rate these similarly?

- PROBE: Both questions reflect an experience of missing out on social interactions due to your CF. In what ways does one question reflect your personal experience more than the other?

*Interview instruction: Highlight the retained question in the slide to show the participant*

- PROBE: Ultimately, we ended up keeping question 23 because responses from survey participants like yourself suggested that feelings of isolation was a more relevant way to describe social limitations than feeling frustrated about missing activities due to CF treatment. What is your reaction to this? Do you agree with this decision?

1. **The last question grouping in this section includes questions 30, 31, and 32.** *Interviewer read questions out loud* **These questions were designed to reflect how your relationships are impacted due to CF. Would you respond to these similarly? Why or why not?**
   - - *PROBE: If respondent needs clarity*: On a 1-5 scale, with 1 being not at all and 5 being an extreme amount, would you rate these similarly?
   - PROBE: All of these questions reflect how personal relationships can be affected by your CF. In your experience, are there differences in the way CF has impacted your relationships with family versus friends?
   - PROBE: Other respondents have responded to questions 31 and 32 similarly. Would you agree that these questions are asking the same thing or is there an important distinction here? Why or why not?

*Interview instruction: Highlight the retained question in the slide to show the participant*

- PROBE: Ultimately, we ended up keeping question 31 because responses from survey participants like yourself suggested that difficult relationships with family members was a more relevant description than the other two aspects of family and friend relationships. What is your reaction to this? Do you agree with this decision?

1. **As we review the final list of questions in this section, both the original and revised question list, we are able to see how many questions have been removed and kept. In this section, we kept 4 of the 8 questions. Is this a meaningful reduction in your mind?**
   - PROBE: Is reducing the time to complete this section worth it to you given the things we may not capture about your CF experience?
   - PROBE: Are there any questions that were removed that you feel should be kept?

**Emotional Domain (Slides 19 – 23)**

*The next section will cover questions about the emotions you may experience due to your CF.*

1. **The first similar pair is questions 11 and 14.** *Interviewer read questions out loud* **These two questions are similar in that they both refer to negative emotions that may arise from someone’s experience with CF. Would you respond to these similarly? Why or why not?**
   - - *PROBE: If respondent needs clarity*: On a 1-5 scale, with 1 being not at all and 5 being extremely, would you rate these similarly?

- PROBE: Do you think one of these questions is better able to reflect your experience with CF? Why or why not?

*Interview instruction: Highlight the retained question in the slide to show the participant*

- PROBE: Ultimately, we ended up keeping question 11 because responses from survey participants like yourself seemed to suggest that feeling angry may be a more relevant way to describe the emotional impact of CF than feeling fearful. What is your reaction to this? Do you agree with this decision?

1. **The next group of similar questions are questions 15, 16, 17, 19.** *Interviewer read questions out loud* **These questions capture mood changes as they relate to your experience with CF? Would you respond to these similarly? Why or why not?**
   - - *PROBE: If respondent needs clarity*: On a 1-5 scale, with 1 being not at all and 5 being extremely, would you rate these similarly?
   - PROBE: What does feeling “frustrated” mean in the context of your CF? Do you think that is the same or different than feeling “irritable” or “moody” or “stressed”? Why or why not?

*Interview instruction: Highlight the retained question in the slide to show the participant*

- PROBE: Ultimately, we ended up keeping question 15 because responses from survey participants like yourself seemed to suggest that feeling frustrated may be a more relevant way to describe the emotional impact of CF than feeling irritable, moody, or stressed. What is your reaction to this? Do you agree with this decision?
  - PROBE: Does “frustrated” capture all of the emotions that were dropped? Why or why not?

1. **The last similar pair in this section is questions 18 and 20.** *Interviewer read questions out loud* **These two questions are intended to cover feelings of unhappiness associated with your CF. Would you respond to these similarly? Why or why not?**
   - - *PROBE: If respondent needs clarity*: On a 1-5 scale, with 1 being not at all and 5 being extremely, would you rate these similarly?

- PROBE: What does feeling “sad” mean in the context of your CF? Do you think that is same or different than feeling “depressed” because of your CF? Why or why not?
- PROBE: Do you think one of these questions is better able to reflect your experience with CF?. Why or why not?

*Interview instruction: Highlight the retained question in the slide to show the participant*

- PROBE: Ultimately, we ended up keeping question 18 because responses from survey respondents like yourself seemed to suggest that feeling sad may be a more relevant way to describe the emotional impact of CF than feeling depressed. What is your reaction to this? Do you agree with this decision?

1. **As we review the final list of questions in this section, both the original and revised question list, we are able to see how many questions have been removed and kept. In this section, we kept 5 of the 10 questions. Is this a meaningful reduction in your mind?**
   - PROBE: Is reducing the time to complete this section worth it to you given the things we may not capture about the impact of CF on your emotions?
   - PROBE: Are there any questions that were removed that you feel should be kept?

**Work/School Limitations Domain (Slides 24 – 26)**

*The last section we will cover in the CF-IQ is work and school limitations. These questions ask about limitations in your professional and academic life and the impact of CF on your long-term goals.*

1. **The only similar pair in this section is questions 37 and 38.** *Interviewer read questions out loud* **These questions are intended to capture the way you feel CF has impacted your future opportunities. Would you respond to these similarly? Why or why not?**
   - - *PROBE: If respondent needs clarity*: On a 1-5 scale, with 1 being not at all and 5 being extremely, would you rate these similarly?
   - PROBE: In your view, what does “goals you can set for your life” mean to you? Do you interpret “goals” to include “job or career choices”? Why or why not?

*Interview instruction: Highlight the retained question in the slide to show the participant*

- PROBE: Ultimately, we ended up keeping question 38 because responses from survey respondents like yourself suggested that impact on future goals was more relevant than the impact on only job or career choices. What is your reaction to this? Do you agree with this decision?

1. **As we review the final list of questions in this section, both the original and revised question list, we are able to see how many questions have been removed and kept. In this section, we kept 3 of the 4 questions. Is this a meaningful reduction in your mind?**
   - PROBE: Is reducing the time to complete this section worth it to you given the things we may not capture about your CF experience?

**SECTION 3: Closing Remarks**

***Time: ~5 minutes***

**Objective:** To capture any final comments, thank the participant for his/her time, explain process for providing payment to participant.

- *We’ve come to the end of the focus group.*
- *Before we wrap up, is there anything else that, in your view, would be important for us to know about the questionnaires we just reviewed and your experience with CF?*

***INTERVIEWER: STOP recording interview now.***

- *If you have any questions about this research after we conclude, please refer to your Informed Consent Form and the contact information for Pharmerit.*
- *RPV will provide you with the payment for your participation in this study in the form of a cash gift card, which we will send to your mailing address.*
- *Thank you very much for your time. We greatly appreciate your participation in this research and all the insights that you provided us today.*
