# Supplementary material for: Psychometric validation of the Cystic Fibrosis Impact Questionnaire (CF-IQ): A patient-reported outcome assessing impacts of cystic fibrosis
Source: PLoS One. 2025 Jan 24;20(1):e0317775. doi: 10.1371/journal.pone.0317775 (PMC11761112; doi:10.1371/journal.pone.0317775)
Supplement: S1 Summary — (DOCX) [file pone.0317775.s002.docx]

**S1 Supplementary Summary: Illustrative example of modern psychometric methods**

### *Detailed examination: physical activity impacts domain*

#### Evaluation of local dependence

The table below displays the six redundant item pairs identified in the Physical Activity Impacts domain via local dependence (LD) statistics. In general, the item with the higher model slope for each pair was retained because the model slope characterizes the precision with which the item measures the domain. An exception was the pair of items 26 and 27, in which item 27 was retained despite having a lower model slope. Item 27 addressed impacts on sleep in general, which was determined to be an important concept to retain during review with both Vertex and the qualitative developer of the Cystic Fibrosis Impact Questionnaire (CF-IQ), Dr Kelly McCarrier; therefore, item 27 was retained in place of item 26.

Redundant pair statistics in the Physical Activity Impacts domain

| **Number** | **Items** | **Chen’s LD statistic *p* values** | **Model slopes** |
| --- | --- | --- | --- |
|  | **Redundant pair** | 0.006 |  |
| 6 | How much of the time did you need to take breaks or rest in order to complete tasks? |  | 2.88^a^ |
| 29 | How much extra help did you need from others because of your CF? |  | 2.56 |
|  | **Redundant pair** | 0.002 |  |
| 26 | Over the past 7 days, how often did your coughing cause you to wake up at night? |  | 1.77^a^ |
| 27 | Over the past 7 days, how often did you get less sleep than you needed because of your CF? |  | 1.61 |
|  | **Redundant pair** | 0.025 |  |
| 3 | Difficulty climbing stairs? |  | 2.58 |
| 4 | Difficulty doing sports/exercise? |  | 2.73 |
|  | **Redundant pair** | 0.006 |  |
| 5 | Difficulty doing housework/yardwork or run errands? |  | 3.40 |
| 26 | Coughing woke you up at night? |  | 1.71^a^ |
|  | **Redundant pair** | 0.031 |  |
| 2 | Difficulty keeping up running? |  | 2.28 |
| 25 | Feeling too sick to attend work/school? |  | 1.93 |
|  | **Redundant pair** | 0.032 |  |
| 2 | Difficulty keeping up running? |  | 2.33^a^ |
| 6 | How much of the time did you need to take breaks or rest in order to complete tasks? |  | 2.86^a^ |

CF, cystic fibrosis; LD, local dependence
^a^The slopes for items 2, 6, and 25 have different values because the model is refit after each item is dropped

Also note, in the above table the slope for item 2 is different because the IRT model was refit after dropping item 25. Doing so slightly modified the estimated item 2 slope from 2.28 to 2.33. Similarly, the slopes for items 6 and 26 shifted after each deletion. At this stage, evidence resulted in the research team retaining item 6. The table below displays seven of 12 items that were retained in the final determination.

The model was reexamined after elimination of redundant items. The final model fit was perfect (root mean square error approximation [RMSEA] = 0.0), and the *p* value increased from 0.29 to 0.46. The internal consistency for this solution, measured by ω, was 0.94. Thus, the removal of redundant items improved the model fit to the retained physical activity domain items, providing further support for dropping these items.

Items retained in the Physical Activity Impacts domain

| **Number** | **Stem** | **Retain** |
| --- | --- | --- |
| 1 | How difficult was it for you to keep up with others while walking? | Yes |
| 2 | How difficult was it for you to keep up with others while running? |  |
| 3 | How difficult was it for you to climb stairs? |  |
| 4 | How difficult was it for you to do physically demanding activities (like exercise or sports)? | Yes |
| 5 | How difficult was it for you to do everyday activities like housework, yardwork, or run errands? | Yes |
| 6 | How much of the time did you need to take breaks or rest in order to complete tasks at home, work, or school? | Yes |
| 7 | How physically strong did you feel? | Yes |
| 25 | Over the past 7 days, how often were you feeling too sick to attend school or work because of your CF? |  |
| 26 | Over the past 7 days, how often did your coughing cause you to wake up? |  |
| 27^a^ | Over the past 7 days, how often did you get less sleep than you needed because of your CF? | Yes |
| 28 | Over the past 7 days, how often did you have a hard time keeping up with your daily tasks (at home, work, or school) because of your CF? | Yes |
| 29 | Over the past 7 days, how much extra help did you need from others because of your CF? |  |

CF, cystic fibrosis
^a^Due to qualitative evaluation results, item 27 was kept instead of item 26, despite item 26 having the better model slope

#### Qualitative adjudication of item redundancy evidence

The CF-IQ item redundancies, determined by LD, were qualitatively assessed for agreement or disagreement on the retained or removed item(s). In the Physical Activity Impacts domain, the following item pairs and set were evaluated:

- Items 3 and 4.
- Items 5 and 26.
- Items 2, 6, 25, and 29
  - Originally this item set was reviewed for LD in pairs (6 and 29, 2 and 25, and 2 and 6). Given the redundancy of LD pairs, these items were reviewed as one item set during the focus group adjudication.

After analyzing the patient agreement and justifications, it was determined that the focus groups supported the LD findings, and the final retained items in the Physical Activity Impacts domain displayed excellent content coverage.

#### Final model and scoring statistics

After finalization of the Physical Activity Impacts domain items, the unidimensional IRT model was refit, and model fit and scoring statistics were computed. The RMSEA fit index was 0, and *p*=0.46, indicating that the fitted model did not fit statistically worse than a perfect-fitting model. Therefore, the unidimensional parameterization was supported. Unweighted scoring was supported by the ω=0.92 statistic, indicating that 92% of the latent score was explained by the unweighted score. This evidence justified assessing reliability and validity on the unweighted score derived from the revised item pool.

Evidence for each of the remaining four domains in the CF-IQ was consistent with those reported in detail for the Physical Activity Impacts domain.
