# Supplementary material for: Psychometric validation of the Cystic Fibrosis Impact Questionnaire (CF-IQ): A patient-reported outcome assessing impacts of cystic fibrosis
Source: PLoS One. 2025 Jan 24;20(1):e0317775. doi: 10.1371/journal.pone.0317775 (PMC11761112; doi:10.1371/journal.pone.0317775)
Supplement: S1 Table — (DOCX) [file pone.0317775.s003.docx]

**S1 Supplementary Table. Known-groups validity at baseline across ppFEV_1_ and CFTRm known groups**

| **Domain** | **Variable** | **Group** | ***n*** | **Group mean** | **Difference from reference (95% CI)^a^** | ***p* value^a^** | **Semipartial ω^2,b^** |
| --- | --- | --- | --- | --- | --- | --- | --- |
| Physical Activity Impacts | ppFEV_1_ | ≤ Median (reference group) | 110 | 21.33 | NA | NA | NA |
|  |  | > Median | 104 | 17.04 | -4.29 (-5.84, -2.74) | <0.001 | 0.118 |
|  | CFTRm use | No use (reference group) | 57 | 20.51 | NA | NA | NA |
|  |  | ≤4 weeks | 3 | 27.67 | 7.16 (0.14, 14.18) | 0.046 | 0.037 |
|  |  | >4 weeks | 154 | 18.61 | -1.90 (-3.74, -0.06) | 0.043 | NA |
| Work/School Limitations Impacts | ppFEV_1_ | ≤ Median (reference group) | 110 | 8.13 | NA | NA | NA |
|  |  | > Median | 104 | 6.79 | -1.34 (-2.11, -0.57) | <0.001 | 0.048 |
|  | CFTRm use | No use (reference group) | 57 | 8.16 | NA | NA | NA |
|  |  | ≤4 weeks | 3 | 11.67 | 3.51 (0.16, 6.86) | 0.040 | 0.043 |
|  |  | >4 weeks | 154 | 7.14 | -1.02 (-1.89, -0.14) | 0.023 | NA |
| Emotional Impacts | ppFEV_1_ | ≤ Median (reference group) | 110 | 10.38 | NA | NA | NA |
|  |  | > Median | 104 | 9.45 | -0.93 (-2.01, 0.15) | 0.090 | 0.009 |
|  | CFTRm use | No use (reference group) | 57 | 10.63 | NA | NA | NA |
|  |  | ≤4 weeks | 3 | 13.33 | 2.70 (-1.94, 7.35) | 0.253 | 0.014 |
|  |  | >4 weeks | 154 | 9.60 | -1.03 (-2.24, 0.19) | 0.097 | NA |
| Social Impacts | ppFEV_1_ | ≤ Median (reference group) | 110 | 7.93 | NA | NA | NA |
|  |  | > Median | 104 | 6.93 | -0.99 (-1.98, -0.01) | 0.048 | 0.014 |
|  | CFTR modulator use | No use (reference group) | 57 | 8.35 | NA | NA | NA |
|  |  | ≤4 weeks | 3 | 12.33 | 3.98 (-0.23, 8.20) | 0.064 | 0.042 |
|  |  | >4 weeks | 154 | 7.01 | -1.34 (-2.44, -0.24) | 0.018 | NA |
| Control and Burden of CF Treatment Impacts | ppFEV_1_ | ≤ Median (reference group) | 110 | 15.17 | NA | NA | NA |
|  |  | > Median | 104 | 13.30 | -1.87 (-3.17, -0.58) | 0.005 | 0.032 |
|  | CFTR modulator use | No use (reference group) | 57 | 15.19 | NA | NA | NA |
|  |  | ≤4 weeks | 3 | 16.00 | 0.81 (-4.86, 6.48) | 0.779 | 0.007 |
|  |  | >4 weeks | 154 | 13.88 | -1.31 (-2.79, 0.17) | 0.083 | NA |

CFTRm, cystic fibrosis transmembrane conductance regulator modulator; CI, confidence interval; NA, not applicable; ppFEV_1_, percent predicted forced expiratory volume in 1 second
^a^*p* values and CIs correspond to the null hypothesis that the effect group is not different from its reference group
^b^Semipartial ω^2^ is a model-based effect size estimator that is less biased than R^2^
